# Supplementary material for: Dignity-based care and infertility treatment: A qualitative study
Source: PLoS One. 2025 Oct 31;20(10):e0334322. doi: 10.1371/journal.pone.0334322 (PMC12578147; doi:10.1371/journal.pone.0334322)
Supplement: Attachment — Submitted filename: PONE-D-25-11046_reviewer.docx [file pone.0334322.s001.docx]

PLOS ONE

**Dignity-based care and infertility treatment: A qualitative study**

--Manuscript Draft--

| **Manuscript Number:** | PONE-D-25-11046 |
| --- | --- |
| **Article Type:** | Research Article |
| **Full Title:** | Dignity-based care and infertility treatment: A qualitative study |
| **Short Title:** | Dignity-based care and infertility treatment: A qualitative study |
| **Corresponding Author:** | Masoumeh Simbar, PhD  Shahid Beheshti University of Medical Sciences School of Nursing and Midwifery Tehran, IRAN, ISLAMIC REPUBLIC OF |
| **Keywords:** | Infertility; dignity-based care; ethics; qualitative study; care |
| **Abstract:** | Introduction  The treatment of infertile people is generally time-consuming and requires frequent and long-term visits and providing dignity-based services. Due to the different perceptions and experiences of people and the lack of a specific study to explain the concept of dignity-based care, this study aimed to explain the concept and dimensions of dignity- based care in infertility treatment services.  Methods  This was a qualitative study with a conventional content analysis approach. Fifty participants (20 infertile women, 16 infertile men, and 14 key informants) were recruited using a purposive sampling method from an educational center of Mazandaran University of Medical Sciences and a private infertility center in Mazandaran –Iran in 2023. Sampling continued until data saturation. Data was collected using in-depth and semi-structured individual interviews. The data were also analyzed using the conventional content analysis method and the steps suggested by Grandheim and Lundman. Also, Lincoln and Guba's criteria were used to check the trustworthiness of the data.  Results  The content analysis demonstrated 43 codes in 11 sub-categories and 4 categories. These categories are “conserving dignity in providing care”, “making the information accessible and obtaining informed consent for care procedure”, “providing professional care and standard services”, and considering cultural and social aspects of infertility. The categories were used to explain the concept and dimensions of dignity-based care in infertility treatment services which are showing the multidimensional aspects of this concept.  Conclusion  Dignity-based care in infertility treatment services means “conserving dignity in providing care services; making the information accessible and obtaining informed consent for care procedure; providing professional care and standard services; and considering cultural and social aspects of infertility.” This concept can be used in future policy-making and planning, and appropriate support should be taken into account to improve the quality of infertility treatment services. |
| **Order of Authors:** | Zahra Kiani |
|  | Masoumeh Simbar, PhD |
|  | Soheila Nazarpour |
|  | Vida Ghasemi |
| **Additional Information:** |  |

| **Question** | **Response** |
| --- | --- |
| **Financial Disclosure**  Enter a financial disclosure statement that describes the sources of funding for the work included in this submission. Review the [submission guidelines](http://journals.plos.org/plosone/s/submission-guidelines#loc-financial-disclosure-statement) for detailed requirements. View published research articles from [*PLOS ONE*](https://journals.plos.org/plosone/) for specific examples.  This statement is required for submission and **will appear in the published article** if the submission is accepted. Please make sure it is accurate. | The author(s) received no specific funding for this work. |
| **Funded studies**  Enter a statement with the following details:   - Initials of the authors who received each award - Grant numbers awarded to each author - The full name of each funder - URL of each funder website - Did the sponsors or funders play any role in the study design, data collection and analysis, decision to publish, or preparation of the manuscript? |  |
| Did you receive funding for this work? |  |
| **Competing Interests**  Use the instructions below to enter a competing interest statement for this submission. On behalf of all authors, disclose any [competing interests](http://journals.plos.org/plosone/s/competing-interests) that could be perceived to bias this work—acknowledging all financial support and any other relevant financial or non- financial competing interests.  This statement is required for submission and **will appear in the published article** if the submission is accepted. Please make sure it is accurate and that any funding sources listed in your Funding Information later in the submission form are also | The authors have declared that no competing interests exist. |

| declared in your Financial Disclosure statement.  View published research articles from  [*PLOS ONE*](http://journals.plos.org/plosone/) for specific examples. |  |
| --- | --- |
| **NO authors have competing interests**  Enter: *The authors have declared that no competing interests exist.*  **Authors with competing interests**  Enter competing interest details beginning with this statement:  *I have read the journal's policy and the authors of this manuscript have the following competing interests: [insert competing interests here]* |  |
| * typeset |  |
| **Ethics Statement**  Enter an ethics statement for this submission. This statement is required if the study involved:   - Human participants - Human specimens or tissue - Vertebrate animals or cephalopods - Vertebrate embryos or tissues - Field research   Write "N/A" if the submission does not require an ethics statement.  General guidance is provided below. Consult the [submission guidelines](http://journals.plos.org/plosone/s/submission-guidelines#loc-guidelines-for-specific-study-types) for detailed instructions. **Make sure that all information entered here is included in the Methods section of the manuscript.** | Include the approval number and/or a statement indicating approval of this research  Study ID, Participant anonymity |

| **Format for specific study types**  **Human Subject Research (involving human participants and/or tissue)**   - Give the name of the institutional review board or ethics committee that approved the study - Include the approval number and/or a statement indicating approval of this research - Indicate the form of consent obtained (written/oral) or the reason that consent was not obtained (e.g. the data were analyzed anonymously)   **Animal Research (involving vertebrate animals, embryos or tissues)**   - Provide the name of the Institutional Animal Care and Use Committee (IACUC) or other relevant ethics board that reviewed the study protocol, and indicate whether they approved this research or granted a formal waiver of ethical approval - Include an approval number if one was obtained - If the study involved *non-human primates*, add *additional details* about animal welfare and steps taken to ameliorate suffering - If anesthesia, euthanasia, or any kind of animal sacrifice is part of the study, include briefly which substances and/or methods were applied   **Field Research**  Include the following details if this study involves the collection of plant, animal, or other materials from a natural setting:   - Field permit number - Name of the institution or relevant body that granted permission |  |
| --- | --- |
|  |  |
| **Data Availability**  Authors are required to make all data underlying the findings described fully available, without restriction, and from the time of publication. PLOS allows rare exceptions to address legal and ethical concerns. See the [PLOS Data Policy](http://journals.plos.org/plosone/s/data-availability) and [FAQ](http://journals.plos.org/plosone/s/data-availability#loc-faqs-for-data-policy) for detailed information. | No - some restrictions will apply |

| A Data Availability Statement describing where the data can be found is required at submission. Your answers to this question constitute the Data Availability Statement and **will be published in the article**, if accepted. |  |
| --- | --- |
| **Important:** Stating ‘data available on request from the author’ is not sufficient. If your data are only available upon request, select ‘No’ for the first question and explain your exceptional situation in the text box. |  |
| Do the authors confirm that all data underlying the findings described in their manuscript are fully available without restriction? |  |
| **Describe where the data may be found in full sentences. If you are copying our sample text, replace any instances of XXX with the appropriate details.**   - If the data are **held or will be held in a public repository**, include URLs, accession numbers or DOIs. If this information will only be available after acceptance, indicate this by ticking the box below. For example: *All XXX files are available from the XXX database (accession number(s) XXX, XXX.*). - If the data are all contained **within the manuscript and/or Supporting Information files**, enter the following: *All relevant data are within the manuscript and its Supporting Information files.* - If neither of these applies but you are able to provide **details of access elsewhere**, with or without limitations, please do so. For example:   *Data cannot be shared publicly because of [XXX]. Data are available from the XXX Institutional Data Access / Ethics Committee (contact via XXX) for researchers who meet the criteria for access to confidential data.*  *The data underlying the results presented in the study are available from (include the name of the third party* | The data that support the findings of this study (including the Transcripts in Farsi and audio files of the interviews) are available from the corresponding author Masoumeh Simbar, but restrictions apply to the availability of these data, which were used under license for the current study, and so are not publicly available. Data are however available from the authors upon reasonable request and with permission of Shahid Beheshti University of Medical Sciences. |

| *and contact information or URL).*   - This text is appropriate if the data are owned by a third party and authors do not have permission to share the data.   * typeset |  |
| --- | --- |
| Additional data availability information: | Tick here if the URLs/accession numbers/DOIs will be available only after acceptance of the manuscript for publication so that we can ensure their inclusion before publication. |

<https://www2.cloud.editorialmanager.com/pone/download.aspx?id=39604646&guid=77b9166b-47cf-4ba5-bb44-33c50480c010&scheme=1>Manuscript	[Click here to access/download;Manuscript;InfertDignity- en2.edited-R2(4).docx](https://www2.cloud.editorialmanager.com/pone/download.aspx?id=39604646&guid=77b9166b-47cf-4ba5-bb44-33c50480c010&scheme=1)

# Title page

**Dignity-based care and infertility treatment: A qualitative study**

Zahra Kiani^1^, Masoumeh Simbar^2*^, Soheila Nazarpour^3^, Vida Ghasemi^4^

^1^ Midwifery and Reproductive Health Research Center, Shahid Beheshti University of Medical Sciences, Tehran, Iran. [Email:zahrakiani6969@gmail.com](mailto:zahrakiani6969@gmail.com)

^2^ Midwifery and Reproductive Health Research Center, Department of Midwifery and Reproductive Health, School of Nursing and Midwifery, Shahid Beheshti University of Medical Sciences, Tehran, Iran. Email: [msimbar@gmail.com](mailto:msimbar@gmail.com)

^3^ Department of Midwifery, Chalous Branch, Islamic Azad University, Chalous, Iran. Email: [sn_9547@yahoo.com](mailto:sn_9547@yahoo.com) ^4^Department	of	Public	Health,	Asadabad	School	of	Medical	Sciences,	Asadabad,	Iran.	Email: [vidaghasemi89@gmail.com](mailto:vidaghasemi89@gmail.com)

**Correspondence**

Masoumeh Simbar, Midwifery and Reproductive Health Research Center, Department of Reproductive Health and Midwifery, School of Nursing and Midwifery, Shahid Beheshti University of Medical Sciences, Vali-Asr Avenue, Cross of Vali-Asr and Neiaiesh Highway, Opposite to Rajaee Heart Hospital, Tehran, Iran. Postal Code: 1996835119; Phone and Fax: (+98) 21-88202512.

Email: [msimbar@gmail.com](mailto:msimbar@gmail.com)

# Dignity-based care and infertility treatment: A qualitative study Abstract

**Introduction:** The treatment of infertile people is generally time-consuming and requires frequent and long-term visits and providing dignity-based services. Due to the different perceptions and experiences of people and the lack of a specific study to explain the concept of dignity-based care, this study aimed to explain the concept and dimensions of dignity-based care in infertility treatment services.

**Methods:** This was a qualitative study with a conventional content analysis approach. Fifty participants (20 infertile women, 16 infertile men, and 14 key informants) were recruited using a purposive sampling method from an educational center of Mazandaran University of Medical Sciences and a private infertility center in Mazandaran –Iran in 2023. Sampling was continued until data saturation. Data were collected using in-depth and semi-structured individual interviews. The data were also analyzed using the conventional content analysis method and the steps suggested by Grandheim and Lundman. Also, Lincoln and Guba's criteria were used to check the trustworthiness of the data.

**Results:** The content analysis demonstrated 43 codes in 11 sub-categories and 4 categories. These categories are “conserving dignity in providing care”, “making the information accessible and obtaining informed consent for care procedure”, “providing professional care and standard services”, and considering cultural and social aspects of infertility. The categories were used to explain the concept and dimensions of dignity-based care in infertility treatment services which are showing the multidimensional aspects of this concept.

**Conclusion:** Dignity-based care in infertility treatment services means “conserving dignity in providing care services; making the information accessible and obtaining informed consent for care procedure; providing professional care and standard services; and considering cultural and social aspects of infertility.” This concept can be used in future policy-making and planning, and appropriate support should be taken into account to improve the quality of infertility treatment services.

**Keywords:** infertility, dignity-based care, ethics, qualitative study, care

# Introduction

Infertility is an important problem in reproductive health. It is defined as not being able to conceive after one year or longer of unprotected sex (1). The trend of infertility in the world is growing in recent years, and according to the latest report of the World Health Organization (WHO), in 2023, the prevalence of periodic and lifetime infertility was 12.6% and 17.5%, respectively (2).

Infertility mostly hurts various dimensions of health and causes a decrease in the couples' quality of life (3). Infertile couples may experience some psychosocial disorders such as anxiety, depression, stress, low self-confidence, social isolation, despair, social stigma, and so on (4, 5).

There are various methods to treat infertility that require frequent visits and high costs of associated treatment (6). Infertile couples do not disclose their infertility, especially in developing countries, and for this reason, sometimes they do not seek treatment (7). So, it is critical to keep human values in consideration, the confidentiality of information, and providing dignity-oriented care to them (3, 4).

Conserving dignity (respect, privacy, effective communication) in providing care services is an approach that is designed based on ethical principles, prioritizes people's preferences, and is based on evidence-based practices (8). Providing respectful care with empathy, support, and by empowering and improving the client's self-confidence are the main features of dignity-based care

(9). In recent years, promoting dignity-based care and considering the cultural, social, and psychological aspects of care are emphasized by national and international organizations (10).

Universal access to sexual and reproductive health services, including infertility treatment services, is accentuated in the sustainable development goals (11). There is a delay in seeking treatment due to the limited access and unaffordability of assisted reproductive technology (ART) in most parts of the world (12). Besides, considering the specific mental conditions of infertile people, preserving human values, paying attention to their expectations, and providing dignity- oriented care in infertility treatment are essential (13-15). However, there is no precise definition of dignity-oriented care in infertility treatment, while addressing it can be very effective on the consequences of infertility and its treatment (16) as well as on the planning for special dignity- oriented services (17).

In a qualitative study on infertile women, Kiani and colleagues emphasized that it is important to follow ethical principles in providing infertility services to reduce the clients’ anxieties (3). Bezad et al. also stated that universal coverage and access to infertility care services is a right and not an option, and providing infertility services along with preserving dignity is an important component of providing infertility services and it should be mentioned and defined as the rights of infertile couples by the health policymakers and program managers (14). Furthermore, Gipson et al., in their review article concluded that infertility is one of the components of sexual and reproductive health and rights that is usually ignored in planning for providing dignity-based services (18).

Although there is a general agreement for dignity-based services, it is ignored in many services and the client's needs are not taken into account(19, 20). The purpose of qualitative research is to understand and explain different people's perceptions and experiences of a phenomenon that usually differ from one person to another (21). Therefore, this qualitative study aimed to explain the concept and dimensions of dignity-based care in infertility treatment services.

**Materials and Methods:**

**Design of the study:** This was a qualitative study with a conventional content analysis approach.

**The participants:** In this qualitative study, infertile men and women and key informants (including specialists in reproductive health, medical ethics, a gynecologist with infertility fellowship, midwives with an experience in infertility services, the health policy maker, as well as an anthropologist, a clinical psychologist and a psychiatrist who had experience with infertile couples. The inclusion criteria for participating in the study include women and men with a one- year history of infertility diagnosed by a gynecologist, with permanent marriage, no adoption, no chronic disease, and no psychiatric disorders. The key informants had at least two years of experience in infertility services, with a minimum Bachelor's degree.

**Sampling:** Purposive sampling method was used for the participants’ recruitment and it was continued until data saturation. All eligible participants accepted to be interviewed and no one refused to participate.

**Setting**: The places of the study were an educational hospital affiliated with Mazandaran University of Medical Sciences as well as a private infertility center in Sari City in April 1, 2023

to December 14, 2023. The interview was performed in a private room in the infertility treatment center. Key information was obtained in their work office.

**The tool for data collection:** The researcher used various methods for data collection including semi-structured interviews, observation, audio recording, writing them down, taking field notes, and document review. The in-depth face-to-face interview began with semi-structured questions such as "What is the meaning of dignity-based care in infertility services?", "What do you expect from dignity-based care in infertility services?” and “Do you receive dignity-based care services?”. All interviews were performed and recorded with the written consent of the participants. The average duration of the interview was around 45 minutes. All people were interviewed once only.

**Trustworthiness:** The trustworthiness of data was analyzed using four criteria suggested by Lincoln and Goba (1994) including credibility, dependability, transferability, and confirmability

(22). Besides, the authenticity of the data was assessed as the fifth criterion that is suggested by Polit and Beck (23).

***Credibility:*** The creditability of data was increased through methods such as (1) prolonged engagement and persistent observation; by spending adequate time for data collection and gaining the trust of the participants, (2) member checking: by asking participants to review and provide feedback on the accuracy of the extracted codes, and to ensure the similarity of the findings with the participants' experiences, (3) peer debriefing: by asking two other researchers to review the research process and finding to ensure objectivity and accuracy of data, (4)Triangulation; using multiple methods and source for data collection such as in-depth individual interview and observation and the literature review, and (5) Improving interview skills: the interviewer (ZK, 32- years-old women, PhD of reproductive health, assistant professor of midwifery and reproductive health research center and infertility researcher) improved her skills by participating in the interview and qualitative content analysis courses.

***Dependability:*** The dependability of the data was increased by asking all the participants questions in the same field, providing a detailed description of the research methods, independent audit and coding of the interviews by two reproductive health outside experts, and also the code-recode method.

***Transferability:*** Transferability of the data was improved by leaving aside the thoughts of the researcher, providing details about the context and the procedure of the study, and agreement of two reproductive health experts on the formation of codes and categories in data analysis.

***Confirmability:*** It was increased by considering maximum diversity in terms of age, education, employment status, type of treatment, and duration of infertility, and providing a rich description of the qualitative method.

***Authenticity:*** It was improved by conveying the feeling tone of infertile couples’ lives by providing vicarious experience, feeling, language, and context of those lives.

**Data Analysis:** Conventional content analysis was performed based on the criteria proposed by Graneheim and Lundman (22). After transcribing the recorded interviews, the text was carefully reviewed by the researcher several times to achieve an accurate understanding of the interview contents. Then, the text was divided into meaning units, meaning units were condensed while preserving the meaning and labeled with codes. Similar codes were then categorized into subcategories, and the subcategories were classified into a category based on common properties. The latent content of the similar categories was finally formulated as a theme.

**Ethics:** The study was approved by the ethical committee of Shahid Beheshti University of Medical Sciences (IR.SBMU.PHARMACY.REC.1401.287). We confirm that all methods were performed in accordance with the relevant guidelines and regulations. Before conducting the interviews, the researcher briefed the participants on the objectives of the study and ensured the confidentiality of their information and the voluntary type of participation. Informed written and verbal consent was also obtained from all participants for the interview and audio recording of the interview.

# Results

Twenty infertile women and 16 infertile men and 14 key informants participated in the study. The demographic characteristics of the participants and the key informants are shown in Tables 1 and 2, respectively.

Table 1. Demographic characteristics of women and men participating in the study “explaining the concept of dignity-based care in infertility treatment services”

| **Characteristics** | **Mean (Women)** | **Standard deviation (Women)** | **Mean (Men)** | **Standard deviation (Men)** |
| --- | --- | --- | --- | --- |

| Age (years) | | 32.16 | 9.68 | 34.58 | 8.79 |
| --- | --- | --- | --- | --- | --- |
| Duration of marriage | | 11.41 | 6.72 | 15.32 | 7.32 |
| Duration of infertility | | 7.82 | 7.95 | 8.05 | 6.45 |
|  | | **Number** | **Percent (%)** | **Number** | **Percent (%)** |
| Education | Elementary | 4 | 20.0 | 2 | 12.5 |
|  | High school | 4 | 20.0 | 3 | 18.75 |
|  | Diploma | 4 | 20.0 | 3 | 18.75 |
|  | Bachelor's degree | 3 | 15.0 | 3 | 18.75 |
|  | Master's degree | 3 | 15.0 | 3 | 18.75 |
|  | Doctorate | 2 | 10.0 | 2 | 12.5 |
| Residence | City | 13 | 65.0 | 9 | 56.25 |
|  | Village | 7 | 35.0 | 7 | 43.75 |
| The economic situation | Low | 6 | 30.0 | 4 | 25.0 |
|  | Medium | 8 | 40.0 | 8 | 50.0 |
|  | High | 6 | 30.0 | 4 | 25.0 |
| Type of infertility | Primary | 12 | 60.0 | 10 | 62.5 |
|  | Secondary | 8 | 40.0 | 6 | 37.5 |
| Type of treatment | Medication | 5 | 25.0 | 4 | 25.0 |
|  | IUI | 5 | 25.0 | 4 | 25.0 |
|  | IVF | 5 | 25.0 | 4 | 25.0 |
|  | Donor eggs | 5 | 25.0 | 4 | 25.0 |

Table 2. The characteristics of women and men participating in the study “explaining the concept of dignity-based care in infertility treatment services”

| **Profession** | **Age (years)** | **Work experience (years)** | **Education** |
| --- | --- | --- | --- |
| Medical ethics | 52 | 29 | PhD |
| Medical ethics | 40 | 10 | PhD |
| Health policy maker | 43 | 13 | PhD |
| Obstetrician gynecologist with infertility fellowship | 55 | 22 | MD |
| Obstetrician gynecologist with infertility fellowship | 46 | 15 | MD |
| Anthropologist | 48 | 20 | PhD |
| Reproductive health specialist | 54 | 21 | PhD |
| Reproductive health specialist | 42 | 10 | PhD |
| Clinical Psychologist | 40 | 11 | PhD |
| Clinical Psychologist | 45 | 20 | BS |
| Psychiatrist | 45 | 17 | MD |
| Midwife | 44 | 16 | BS |
| Midwife | 37 | 12 | MS |
| Midwife | 31 | 7 | MS |

The content analysis of the data demonstrated 43 codes, 11 categories, and 4 categories (Table 3).

Table 3. The codes, subcategories and categories of the concept dignity-based care in infertility treatment services

| **Categories** | **Categories** | **Codes** |
| --- | --- | --- |
| Conserving dignity in providing care | Respect | Introducing of the clinic personnel and their tasks |
|  |  | Providing patient‐friendly clinic environments |
|  |  | Providing proper and on time services |
|  |  | Providing welfare facilities |
|  |  | Avoiding discrimination in providing the services |
|  | Privacy | Providing private room for counseling, physical exams and  care procedures |
|  |  | Absence of unnecessary attendees during counseling and care  procedures |
|  |  | Considering confidentiality of the information |
|  | Effective communication | Effective verbal communication |
|  |  | Effective non-verbal communication |
|  |  | Providing couples’ counseling services |
| Access to information and considering informed consent | Making Awareness | Providing the necessary information about the infertility  problem to the couples |
|  |  | Explaining about the different treatment methods |
|  |  | Providing information about the procedure of the treatment |
|  |  | Informing the couples about the success rate |
|  |  | Providing information about the costs |
|  |  | Awareness about treatment repeatability |
|  | Freedom to choose | Avoiding coercion for treatment time |
|  |  | Giving adequate information to make correct decision |
|  |  | Giving freedom to choose the treatment team |
|  | Participatory decision making | Considering the couples’ needs, expectations and demands |
|  |  | Obtaining permission to perform care procedures |
|  |  | Providing a couple-friendly atmosphere for making decisions  about treatment |
| Providing professional care and standard services | Professional care | Providing a team-work services |
|  |  | Providing mental care |
|  |  | The availability of the treatment team |
|  |  | Performing care procedures based on the protocols |
|  | Standard services | Avoiding unnecessary interventions |
|  |  | Providing on time services |
|  |  | Increasing the number of public infertility centers |
|  |  | Providing adequate infertility specialists |
|  |  | Providing appropriate insurance coverage |
|  |  | Government financial support |
| Considering the cultural and social aspects of infertility | Attention to the common beliefs of society | Children as the life support |
|  |  | Difficulty in acceptance of couples’ life without kids |
|  |  | Considering infertility as a female issue |
|  |  | Sterility stigmatization |
|  | Modification of misbeliefs and attitudes | Considering the family education |
|  |  | The Community education |
|  |  | Involving national media |
|  | recruiting the  eligible personnel | Providing native treatment team |
|  |  | Treatment by a same-sex care provider |

|  |  | Training of non-native treatment team |
| --- | --- | --- |

# 1 Conserving dignity in providing care

This theme consists of three categories, respect, privacy, and effective communication.

# 1-1 Respect

All of the participants emphasized providing respectful care as an important approach to providing dignity-based care, through performing procedures such as Introducing the clinic personnel and their tasks, providing patient-friendly clinic environments, providing proper and on-time services, providing welfare facilities, and avoiding discrimination in providing the services.

*"When I arrived in the clinic, a lady in charge introduced herself to us and explained about the services for infertile couples in the clinic. I felt relaxed and I thought that she respects me and my husband" (32-year-old woman, housewife, 8-year history of primary infertility).*

*“Sometimes we wait a lot here. I wish the timings were more precise. This is a way and a meaning for providing respectful care" (45-year-old man, 15-year history of primary infertility).*

*"I came to the center from the nearby cities for treatment and it is difficult for me to travel between the cities. I wish the center had a place for us to stay, where we would reserve the place in advance so that we don't have to worry about our accommodation. In this way, one feels more respected" (38-year-old woman, housewife, 10-year history of secondary infertility).*

# 1-2 Privacy

The majority of participants stated that providing privacy in the care procedure is a critical item for dignity-based care. They mentioned providing a private room for counseling, physical exams, and care procedures; the absence of unnecessary attendees during counseling and care procedures; and considering the confidentiality of the information.

*"My wife and I expect that the infertility center keeps our information a secret. Sometimes my mother insists on coming with us, but I don't want her to come with us, and I like that even when*

*she comes with us, I expect the doctor allows no one to be there except me and my wife “(37- years-old man, 5-years history of primary infertility).*

*"I don't like that our personal information such as the cause of our infertility, the treatment procedures, and the treatment repetition, to be revealed to anyone; as these are from our private issues" (32-year-old woman, housewife, 12-year history of infertility).*

# 1-3 Effective communication

All participants mentioned effective verbal and non-verbal communication in providing couple's counseling procedures.

*“Couples often say that providing couple’s counseling with effective communication help in improving marital relationship, and they suppose that their dignity is being preserved” (Clinical Psychologist, 20 years of work experience).*

*"It is very important to me that when I talk to the doctor or the treatment personnel, they listen to me carefully and sometimes confirm my words by shaking their head or with their eyes" (26- year-old woman, housewife, 5-year history of primary infertility).*

# 2 Access to information and considering informed consent

This theme is extracted as a significant dimension of the concept of dignity-based care in infertility services which means making awareness, freedom of choice, and participatory decision-making.

# 2-1 Making awareness

The majority of participants mentioned making awareness as an important factor in providing dignity-based care in infertility services. This category includes the codes such as providing the necessary information about the infertility problem to the couples; explaining the different treatment methods; providing information about the procedure of the treatment; informing the couples about the success rate; and providing information about the costs.

*"It is very important to be aware of different methods of infertility treatment and the treatment personnel should explain this to us" (33-year-old woman, housewife, 15-year history of primary infertility).*

*"I would like the doctor to explain to me about the chance of the treatment for example by IVF" (40-year-old woman, employed, 7-year history of secondary infertility).*

*“Some doctors use English words, for example, they say IVF or IUCS? I wish they could tell us in simple language what these are?” (33 years old man, 6-years history of primary infertility).*

*"I think it is very important to know how likely different methods could be successful in treating infertility and to have this information at our disposal" (45-year-old woman, housewife, 20-year history of primary* infertility).

# 2-2 Freedom to choose

The majority of participants emphasized avoidance of coercion for treatment time; giving adequate information to make correct decisions; and giving freedom to choose the treatment team as the means for providing dignity-based care services.

*"I like to be informed about all the procedures that should be provided for me with details and take my consent before performing the procedure." (37-year-old man, 5-years history of primary infertility).*

*"I am an employee and I came here from another province for treatment and it was difficult for me to take time off. I would like that they ask me when it is possible for me, rather than making an appointment by themselves" (45-year-old woman, Employed, 12-year history of primary infertility).*

*"I can't communicate with some doctors. I wish it was possible to choose the doctor and treatment team ourselves" (33-year-old woman, housewife, 16-year history of primary infertility).*

# 2-3 Participatory decision making

The participants mentioned considering couples' needs, expectations, and demands; obtaining permission to perform any care procedures, and providing a couple**-**friendly atmosphere for making decisions about treatment as the meaning of participatory decision-making.

*“I think we should have a role in making decisions about any care that we receive, and we should be able to talk about that easily” (40-year-old woman, employed, 9-years history of primary infertility).*

*"In my opinion, we should be consulted and informed before any care procedure and take our permission first" (33 years old man, 6-year history of primary infertility).*

*"The doctor and other personnel of infertility centers should create an appropriate atmosphere where people can express their wishes about treatment (psychiatrist, 17 years of work experience).*

# 3 Providing professional care and standard services

Providing professional care and standard services was explained as the most important meaning of providing dignity-based care services.

# 3-1 Professional care

Many participants described providing professional care as a critical aspect of dignity-based care services that can be demonstrated by providing a teamwork service; availability of the treatment team; providing mental care; and performing care procedures based on the protocols.

*"The treatment personnel should be a team that should also be available to answer the questions, solve the problems and concerns" (30-year-old man, 3-years history of primary infertility).*

*“One of the important aspects of infertile couples’ care is attention to their psychological changes that should be considered in providing the professional care” (medical ethics specialist, 29-years history of work experience).*

# 3-2 Standard services

Several participants talked about providing standard services as a dignity-based care service which means avoiding unnecessary interventions; providing on-time services; increasing the number of public infertility centers; providing adequate infertility specialists; providing appropriate insurance coverage; and providing financial support by the government.

*“The training of the specialized infertility treatment staff is one of the important requirements in providing dignity-oriented infertility services" (Infertility fellow, 22 years of work experience).*

*“Sometimes I feel that the lack of financial support and appropriate insurance for infertility treatment makes us deprived of standard care treatment (28-year-old woman, working, 5 years of primary infertility history).*

*"Considering the recent policy of rejuvenating the population in the country, one of whose strategies is to help with the treatment of infertile couples, it is necessary to provide public infertility treatment services, with sufficient teams of specialists and experienced personnel and with an appropriate financial support or insurance." (A reproductive Health specialist with, 21- years of work experience).*

# 4 Considering the cultural and social aspects of infertility

Considering the cultural and social aspects of infertility is an important dimension for providing dignity-based services. This approach can be achieved by a few strategies such as attention to the common beliefs of society; modification of misbeliefs and attitudes; and recruiting eligible personnel.

# 4-1 Attention to the common beliefs of society

The majority of participants stressed some common misbeliefs in society that are necessary to be considered in dignity-based care services, such as considering children as the support of life; or difficulty in accepting couples' life without kids; considering infertility as a female's problem; and sterility stigmatization.

*“In traditional societies, a woman's identity is completed when she has children” (anthropologist, 20 years of work experience).*

*“Some people in our society still consider the child as a life supporter.” (A reproductive Health specialist with, 21-years of work experience).*

*It is difficult for the community to accept child-free families. These couples may feel stigmatized. (A reproductive Health specialist with, 10-years of work experience).*

*"Most people think that the problem of infertility is only related to women" (45-year-old woman, housewife, 12-year history of secondary infertility).*

# 4-2 Modification of misbeliefs and attitudes

All Participants emphasized the critical role of a dignity-based service in modifying misbeliefs. They told that family and community education, as well as national media involvement for this modification, are necessary.

*“I wish families would be taught in the infertility center that my wife and I are undergoing treatment and there is no need for their interference” (33-year-old woman, employed, 7-year history of primary infertility).*

*“One of the important issues in the field of infertility care is educating the family so that their beliefs and attitudes can be corrected” (Reproductive health specialist, 10 years of work experience).*

*"In my opinion, the dignity-based infertility treatment centers should attempt to involve national media for correcting the misbeliefs about infertility in the community” (anthropologist, 20 years of work experience).*

# 4-3 Recruiting eligible personnel

The majority of personnel mentioned their preference in providing care by a native treatment team; by a same-sex care provider, and by trained nonnative personnel.

*“When the treatment provider is from our local area, I feel it is easier to understand our words and concerns" (27-year-old woman, housewife, 4-year history of secondary infertility).*

*“The native treatment personnel can understand well how to look at us as an infertile couple in the family and the community (36-year-old woman, housewife, 13-year history of primary infertility).*

# The concept of dignity-based care in infertility treatment services

The concept of "Dignity-based care in infertility treatment services" is conserving dignity (respect, privacy, effective communication) in providing care services; making the information accessible and obtaining informed consent for care procedures; providing professional care and standard services; with considering cultural and social aspects of infertility (Figure 1).

# Discussion

This was the first study to explain the concept of dignity-based care in infertility treatment services. The study demonstrated the concept with four dimensions including conserving dignity in providing care; making the information accessible and obtaining informed consent for care procedure; providing professional care and standard services; with considering cultural and social aspects of infertility. Immanuel Kant was one of the first people who defined dignity as a quality belonging to all sentient human beings because of their capacity for rationality and moral freedom (24), but generally, the concept of dignity is complex and ambiguous (25). According to Kant’s principles, if a person is deprived of the capacity for rational and moral self-determination (e.g., in a Persistent vegetative state, in a coma, etc.), they lack dignity while Aquinas considers dignity to be the fundamental perfection of man that distinguishes him from other beings (things), therefore, as long as a human being exists, they have a dignity proper to themselves, regardless of changing qualities. Hence, this can be called the existential dignity (26). Nowadays, many researchers are trying to explain the concept and dimensions of dignity in different areas of healthcare system as it is known as an Inalienable right of patients (27, 28).

Conserving dignity in providing care was found as a dimension of dignity-based care in infertility treatment services that can be achieved by providing respectful care, keeping the clients' privacy, and making effective communication between the providers and the clients. Conserving human dignity is at the center of moral theories (29) and it is an intrinsic value that every human being seeks and likes to experience (30). The options for infertility treatment range from providing simple counseling to invasive medical interventions, and the infertile clients must provide personal and family information including a detailed history of their physical, mental, and reproductive health condition, as well as the results of biochemical-, Genetic- and screening tests and also imaging. They may be recommended for accepting sperm or egg donation, or surrogacy. Therefore, providing respectful care and counseling, with keeping the clients’ privacy and confidentiality of the information is critical (31). On the other hand, they generally wish their information about the cause of infertility, the course of treatment, and the number of treatment cycles to be kept confidential (32), because they feel that disclosing the information increase the family and social pressure on them and also their privacy will be in threat (33). Since pregnancy is a critical aim in marital life, infertile couples experience extensive changes in their health

especially mental health (34). Therefore, effective communication between infertile couples with the treatment provider is essential to conserve the clients’ dignity (15).

The results demonstrated that making the information accessible for the clients and obtaining informed consent is essential for dignity-based care in infertility treatment services. This aspect of dignity care is achievable by providing the necessary information about infertility, the different treatment methods, the procedure of the treatment, the success rate, and also the costs. In a qualitative study by Kiani et al., the majority of participants stated that they would like to receive detailed information about the different methods of treatments, the repeatability of the treatments, and their rate of success (3). The Council for International Organizations of Medical Sciences (CIOMS) defines “informed consent” as (1) receiving the necessary information to make an informed choice, (2) understanding the information, and (3) making a voluntary decision(35). Besides, in the Belmont Report by the National Commission for the Protection of Human Subjects of Biomedical and Behavioral Research, the importance of providing information, obtaining informed consent, and making independent care decisions was emphasized (36). Failure to give necessary information to the clients reduces the trust in providers and make a negative effect on the receiving treatment and the necessary follow-up (37). In a qualitative study, women stated that they did not receive the necessary information in their treatment procedure and did not participate in making the decisions and so their dignity was not conserved, and so they decided to discontinue the treatment (38).

Providing professional care and standard services was found to be another criterion for providing dignity-based infertility treatment services. In the International Conference of Population and Development in Cairo in 1994, prevention and appropriate treatment of infertility was emphasized as an important health priority, but unfortunately, it is still a great dilemma in the health systems of many countries (39). Unaffordable treatment options, inadequate funds, and experts especially in public centers are the most common problems in providing infertility treatment services (40). In a qualitative study, the participants mentioned the importance of accessible, continuous, and coordinate care with minimized cost for providing patient-centered care in infertility standard treatment services (41). The current infertility policies cause an inappropriate distribution of public and private centers (42). Financial problems in providing medicine and infertility treatment and inappropriate coverage of insurance requirements are to be solved in providing infertility care services (43).

The finding showed that considering the cultural and social aspects of infertility is an important dimension for providing dignity-based services. A few strategies such as attention to the common beliefs of society, modification of misbeliefs and attitudes, and recruiting eligible personnel were suggested. In Iranian and Islamic culture children are considered as a gift. Besides, it is difficult for the community to understand couples’ life without kids, and childlessness is considered stigmatization which is called "stove blindness" (44, 45). In a qualitative study using social constructivism theory, Hasanpour et al demonstrated that infertile couples face several mental and psychological problems that are created by society and are the product of the complex interaction of social relations, expectations, women's needs, the definition of infertility by society, and the view of society towards infertile people (46). The necessity for educating the family and community for making proper beliefs and behaviors about infertility and infertile couples is emphasized in several studies (47-51). Consistent with our findings Sami and Saeed Ali showed that women prefer to be treated by the same sex care providers and local personnel who are familiar with the infertility-related culture in that region because they can understand the infertility cultural problems (38).

# Limitation and strength

The main limitation of this study similar to any other qualitative study, is the non-generalizability of the findings. The strength of the study is the explanation of the concept of dignity-based care in infertility treatment services using the perceptions of infertile couples and the key informants with diverse characteristics.

# Conclusion

Dignity-based care in infertility treatment services means “conserving dignity (respect, privacy, effective communication) in providing care services; making the information accessible and obtaining informed consent for care procedure; providing professional care and standard services; and considering cultural and social aspects of infertility.” This concept can be used in future policy-making and planning, and appropriate support should be taken into account to improve the quality of infertility treatment services.

# Acknowledgments

The authors express their gratitude to women participating in the research, employees of infertility centers and all the researchers whose articles were analyzed, and the Nursing and Midwifery Faculty of the University of Medical Sciences for their support and contribution to this work.

# References

.1

2020.

.2

.3

Berek JS. Berek & Novak’s Gynecology Essentials: Lippincott Williams & Wilkins;

World Health Organization . Infertility prevalence estimates: 1990–2021. 2023.

Kiani Z, Simbar M, Hajian S, Zayeri F. Quality of life among infertile women living in a

paradox of concerns and dealing strategies: a qualitative study. Nursing Open. 2021;8(1):251-61.

.4 Kiani Z, Simbar M, Hajian S, Zayeri F, RashidiFakari F, Chimeh FJ. Investigating

different dimensions of infertile women’s quality of life: a descriptive cross-sectional study. BMC Public Health. 2022;22(1):1-11.

.5 Kiani Z, Simbar M. Infertility’s Hidden and Evident Dimensions: A Concern Requiring

Special Attention in Iranian Society. Iranian Journal of Public Health. 2019;48(11):2114.

.6 Maroufizadeh S, Karimi E, Vesali S, Samani RO. Anxiety and depression after failure of

assisted reproductive treatment among patients experiencing infertility. International Journal of Gynecology & Obstetrics. 2015;130(3):253-6.

.7 Hasanpoor-Azghdy SB, Simbar M, Vedadhir A. The social consequences of infertility

among Iranian women: a qualitative study. International journal of fertility & sterility. 2015;8(4):409.

.8 Agnelli R, Skalko J. Human dignity. Ethics & Medics. 2011.
.9 Thompson GN, Chochinov HM. Dignity-based approaches in the care of terminally ill

patients. Current opinion in supportive and palliative care. 2008;2(1):49-53.

.10 Scarffe E. Toward a Dignity-Based Account of International law. Jus Cogens.

2022;4(3):207-36.

.11 FUND S. Sustainable development goals. 2015.
.12 Banker M, Dyer S, Chambers GM, Ishihara O, Kupka M, de Mouzon J, et al.

International Committee for Monitoring Assisted Reproductive Technologies (ICMART): world report on assisted reproductive technologies, 2013. Fertility and Sterility. 2021;116(3):741-56.

.13 Kiani Z, Simbar M, Dolatian M, Zayeri F. Women’s empowerment in reproductive

decision-making needs attention among Iranian women. Iranian journal of public health. 2018;47(3):464.

.14 Bezad R, Omrani SE, Benbella A, Assarag B. Access to infertility care services towards

Universal Health Coverage is a right and not an option. BMC Health Services Research. 2022;22(1):1089.

.15 Serour GI, Serour AG. Ethical issues in infertility. Best practice & research Clinical

obstetrics & gynaecology. 2017;43:21-31.

.16 Ombelet W. Global access to infertility care in developing countries: a case of human

rights, equity and social justice. Facts, views & vision in ObGyn. 2011;3(4):257.

.17 Ombelet W. WHO fact sheet on infertility gives hope to millions of infertile couples

worldwide. Facts, Views & Vision in ObGyn. 2020;12(4):249.

.18 Gipson JD, Bornstein MJ, Hindin MJ. Infertility: a continually neglected component of

sexual and reproductive health and rights. Bulletin of the World Health Organization. 2020;98(7):505.

.19 Ayoubi S, Pazandeh F, Simbar M, Moridi M, Zare E, Potrata B. A questionnaire to assess

women's perception of respectful maternity care (WP-RMC): Development and psychometric properties. Midwifery. 2020;80:102573.

.20 Freedman LP, Kruk ME. Disrespect and abuse of women in childbirth: challenging the

global quality and accountability agendas. The Lancet. 2014;384(9948):e42-e4.

.21 Ritchie J, Lewis J, Nicholls CM, Ormston R. Qualitative research practice: A guide for

social science students and researchers: sage; 2013.

.22 Lindgren B-M, Lundman B, Graneheim UH. Abstraction and interpretation during the

qualitative content analysis process. International journal of nursing studies. 2020;108:103632.

.23 Polit D, Yang F. chapter5. Measurement and the measurement or change: a primer for the

health professions Philadelphia: Wolters Kluwer. 2016:5.

.24 Sensen O. Kant's conception of human dignity. 2009.
.25 Moody HR. Why dignity in old age matters. Journal of Gerontological Social Work.

1998;29(2-3):13-38.

.26 Ferdynus MP. Is Dignity Still Necessary in Health Care? From Definition to Recognition

of Human Dignity. Journal of religion and health. 2024;63(2):1154-77.

.27 Lin Y-P, Watson R, Tsai Y-F. Dignity in care in the clinical setting: a narrative review.

Nursing ethics. 2013;20(2):168-77.

.28 Barclay L. In sickness and in dignity: a philosophical account of the meaning of dignity

in health care. International journal of nursing studies. 2016;61:136-41.

.29 Jones DA. Human dignity in healthcare: a virtue ethics approach. The New Bioethics.

2015;21(1):87-97.

.30

302.

.31

Jacobson N. Dignity and health: a review. Social science & medicine. 2007;64(2):292-

Wells KJ, Gordon JR, Su HI, Plosker S, Quinn GP. Ethical considerations for informed

consent in infertility research: The use of electronic health records. Advances in medical ethics. 2015;2.

.32 Zargham-Boroujeni A, Jafarzadeh-Kenarsari F, Ghahiri A, Habibi M. Empowerment and

sense of adequacy in infertile couples: a fundamental need in treatment process of infertility-a qualitative study. The Qualitative Report. 2014;19(6):1.

.33 Minucci D. Psychological and ethical implications related to infertility. International

Journal of Gynecology & Obstetrics. 2013;123:S36-S8.

.34 Patel A, Sharma P, Kumar P. Role of mental health practitioner in infertility clinics: A

review on past, present and future directions. Journal of Human Reproductive Sciences. 2018;11(3):219.

.35 Sciences CfIOoM. International ethical guidelines for biomedical research involving

human subjects. Bulletin of medical ethics. 2002(182):17-23.

.36 Louisell DW. National Commission for the Protection of Human Subjects of Biomedical

and Behavioral Research: Research on the Fetus. Vill L Rev. 1977;22:315.

.37 Covington SN. Infertility counseling in practice: a collaborative reproductive healthcare

model. Infertility Counseling: A Comprehensive Handbook for Clinicians (Eds SN Covington, LH Burns). 2006:493-507.

.38 Sami N, Saeed Ali T. Perceptions and experiences of women in Karachi, Pakistan

regarding secondary infertility: results from a community-based qualitative study. Obstetrics and gynecology international. 2012;2012.

.39 Widge A, Cleland J. The public sector's role in infertility management in India. Health

Policy and Planning. 2009;24(2):108-15.

.40 Nachtigall RD. International disparities in access to infertility services. Fertility and

sterility. 2006;85(4):871-5.

.41 Webair HH, Ismail TAT, Ismail SB, Khaffaji AJ. Patient-centred infertility care among

Arab women experiencing infertility: a qualitative study. BMJ open. 2021;11(6):e044300.

.42 Zuccala E, Horton R. Addressing the unfinished agenda on sexual and reproductive

health and rights in the SDG era. The Lancet. 2018;391(10140):2581-3.

.43 Morshed-Behbahani B, Lamyian M, Joulaei H, Montazeri A. Analysis and exploration of

infertility policies in Iran: A study protocol. Health Research Policy and Systems. 2020;18(1):1- 7.

.44 Bokaie M, Simbar M, Ardekani SMY. Sexual behavior of infertile women: a qualitative

study. Iranian journal of reproductive medicine. 2015;13(10):645.

.45 Abbasi-Shavazi MJ, McDonald P. Fertility and multiculturalism: Immigrant fertility in

Australia, 1977–1991. International Migration Review. 2000;34(1):215-42.

.46 Hasanpoor-Azghdy SB, Simbar M, Vedadhir A. The emotional-psychological

consequences of infertility among infertile women seeking treatment: Results of a qualitative study. Iranian journal of reproductive medicine. 2014;12(2):131.

.47 Kura S, Vince J, Crouch-Chivers P. Male involvement in sexual and reproductive health

in the Mendi district, Southern Highlands province of Papua New Guinea: a descriptive study. Reproductive health. 2013;10(1):1-10.

.48 Hasanpoor-Azghady SB, Simbar M, Vedadhir AA, Azin SA, Amiri-Farahani L. The

social construction of infertility among Iranian infertile women: a qualitative study. Journal of reproduction & infertility. 2019;20(3):178.

.49 Yao H, Chan CHY, Chan CLW. Childbearing importance: A qualitative study of women

with infertility in China. Research in Nursing & Health. 2018;41(1):69-77.

.50 Alamin S, Allahyari T, Ghorbani B, Sadeghitabar A, Karami MT. Failure in identity

building as the main challenge of infertility: a qualitative study. Journal of reproduction & infertility. 2020;21(1):49.

.51 Batool SS, de Visser RO. Experiences of infertility in British and Pakistani women: A

cross-cultural qualitative analysis. Health Care for Women International. 2016;37(2):180-96.

Figure	[Click here to access/download;Figure;Figure 1.docx](https://www2.cloud.editorialmanager.com/pone/download.aspx?id=39530556&guid=fff7017a-7ee1-4eeb-b3e7-9e5d65b42e98&scheme=1)[<https://www2.cloud.editorialmanager.com/pone/download.aspx?id=39530556&guid=fff7017a-7ee1-4eeb-b3e7-9e5d65b42e98&scheme=1>](https://www2.cloud.editorialmanager.com/pone/download.aspx?id=39530556&guid=fff7017a-7ee1-4eeb-b3e7-9e5d65b42e98&scheme=1)

- Respect

- Privacy
- Effective communication
- Awareness
- Freedom of choice
- Participatory decision making

Conserving dignity in providing care

# Access to information and considering informed consent

- Professional care
- Standard services

# Providing professional care and standard services

**Considering cultural and social aspects of infertility**

o the common ociety

• Attention t beliefs of s

• Modificati and attitud

• Recruiting treatment t

on of misbeliefs es

eligible eam

Figure 1. The concept of dignity-based care in infertility treatment services

[Click here to access/download](https://www2.cloud.editorialmanager.com/pone/download.aspx?id=39530557&guid=eb2e1bf0-6da1-469a-ac61-71198a10a932&scheme=1)

[**Supporting Information** TheGuideQuestions.docx](https://www2.cloud.editorialmanager.com/pone/download.aspx?id=39530557&guid=eb2e1bf0-6da1-469a-ac61-71198a10a932&scheme=1)
